# The impact of West Nile virus on the abundance of selected North American birds

**DOI:** 10.1186/1746-6148-7-43

**Published:** 2011-08-11

**Authors:** Ivo M Foppa, Raphaelle H Beard, Ian H Mendenhall

**Affiliations:** 1 Department of Epidemiology, Tulane School of Public Health and Tropical Medicine, New Orleans, Louisiana, USA; 2 Dept of Emergency Medicine, Johns Hopkins University School of Medicine, Baltimore, Maryland, USA; 3 Department of Tropical Medicine, Tulane School of Public Health and Tropical Medicine, New Orleans, Louisiana, USA; 4 Program in Emerging Infectious Diseases, Duke-NUS Graduate Medical School, Singapore

## Abstract

**Background:**

The emergence of West Nile virus (WNV) in North America has been associated with high mortality in the native avifauna and has raised concerns about the long-term impact of WNV on bird populations. Here, we present results from a longitudinal analysis of annual counts of six bird species, using North American Breeding Bird Survey data from ten states (1994 to 2010). We fit overdispersed Poisson models to annual counts. Counts from successive years were linked by an autoregressive process that depended on WNV transmission intensity (annual West Nile neuroinvasive disease reports) and was adjusted by El Niño Southern Oscillation events. These models were fit using a Markov chain Monte Carlo algorithm.

**Results:**

Model fit was mostly excellent, especially for American Crows, for which our models explained between 26% and 81% of the observed variance. The impact of WNV on bird populations was quantitatively evaluated by contrasting hypothetical count trajectories (omission of WNV) with observed counts. Populations of American crows were most consistently affected with a substantial cumulative impact in six of ten states. The largest negative impact, almost 60%, was found in Illinois. A regionally substantial decline was also seen for American Robins and House Sparrows, while the other species appeared unaffected.

**Conclusions:**

Our results confirm findings from previous studies that single out American Crows as the species most vulnerable to WNV infection. We discuss strengths and limitations of this and other methods for quantifying the impact of WNV on bird populations.

## Background

In 1998, unusual mortality in Domestic Geese (*Anser anser*) and White Storks (*Ciconia ciconia*) in Israel was attributed to WNV infection [1,2]. These were the first reports of lethal WNV infection in birds outside the laboratory. The following year, widespread mortality in wild and exotic birds became the key signature of WNV emergence in North America [3]. The WNV variant in North America was closely related to the virus that had been identified in the epizootic in Israel [4]. American Crows (*Corvus brachyrhynchos*), as well as other members of the crow family (Corvidae), such as Blue Jays, suffered high mortality [5-10]. Crow mortality was proposed as a reliable indicator of WNV activity [11-17]. Like their old-world relatives [18], American Crows invariably succumbed to experimental WNV infection [19,20]. Directly monitored populations of these birds suffered catastrophic mortality due to WNV infection [21,22]. These observations spawned concerns about the threat WNV might pose to the North American avifauna. While only individual monitoring can offer direct insight into the impact of WNV on bird populations, this approach is impractical for assessing changes on large temporal and geographic scales. Statistical analysis of count data from well-established national or international bird surveillance programs, such as the North American Breeding Bird Survey (BBS), may offer clues on the impact of WNV on seasonal bird populations. Any systematic and substantial demographic impact of WNV on birds should be noticeable in such surveys. Established in 1966 [23], the BBS collects annual abundance data on over 420 bird species along more than 4,100 survey routes, each 24.5 miles long. These routes are primarily surveyed in June [23], coinciding with early seasonal WNV transmission.

Several studies have analyzed the impact of WNV on bird abundance using BBS data. Fujisaki et al. [24], for example, used change point analysis to detect abrupt population changes in bird populations and used the decline of counts of American Crows in Maryland and Virginia in the wake of WNV emergence as a case study. While the resulting change point estimates accurately reflect the time when WNV emerged in these states, the method does not lend itself to impact quantification. Koenig et al. [25] found a significant decline in corvid species between 2004 and 2005, concurrent with widespread transmission in California.

LaDeau et al. [26] used an extrapolation method to estimate the impact of WNV on birds. They fit over-dispersed Poisson models to 26 years of BBS count data, adjusting for observer effects and macro-climatological factors. Based on the resulting parameter estimates and covariate profiles after WNV emergence, they constructed hypothetical count trajectories that might have been observed in the absence of WNV and could be contrasted with observed counts. This method found a significant decline in seven species of birds and estimated a regional decline of American Crows by 45%. Wheeler et al. [27] used the same method, in combination with seroprevalence, dead bird surveillance and susceptibility data and found that, besides American Crows, populations of House Finches (*Carpodacus mexicanus*), Black-crowned Night Herons (*Nycticorax nycticorax*), Western Scrub Jays (*Aphelocoma californica*) and Yellow-billed Magpies (*Pica nuttalli*) were particularly affected by WNV. These studies, however, did not take into account temporal autocorrelation of bird counts that results from the fact that one year's population includes some of previous year's individuals as well as previous year's offspring. An autoregressive model may therefore be more appropriate for the quantification of the impact of WNV than a model that ignores this dependence. The purpose of our study was to quantify the impact of WNV on six North American bird species using an autoregressive approach. First, we fit autoregressive overdispersed Poisson models to annual bird counts that included an indicator of WNV transmission intensity. This type of model is intuitive, as it accounts for the underlying demographic process. Year-to-year changes in bird populations due to WNV are likely mediated by WNV-associated mortality. Using parameter estimates obtained by fitting such models, but omitting terms associated with WNV transmission, we then constructed counterfactual (hypothetical) count trajectories that can be compared to observed counts. The difference between hypothetical and observed counts is interpreted as impact due to WNV. We compare the resulting impact estimates to estimates obtained by two other methods, including one similar to the method used by LaDeau et al [26].

## Results and Discussion

### Model fit and impact estimates

**Figure 1:**
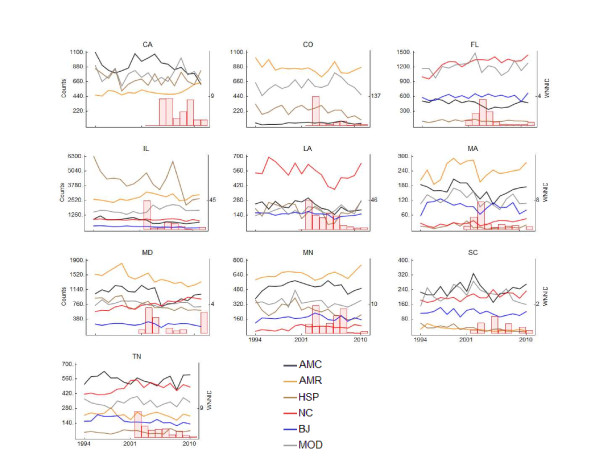
**Annual BBS counts of six species of birds on ten states and annual WNNID reports, by state**. The color of the lines that connect counts indicates bird species: AMC = American Crows; AMR = American Robins; HSP = House Sparrows; NC = Northern Cardinals; BJ = Blue Jays; MOD = Mourning Doves. The tick mark on the secondary y-axis indicates the maximum annual incidence per 1,000,000 of WNNID in a state (red bars).

**Figure 2:**
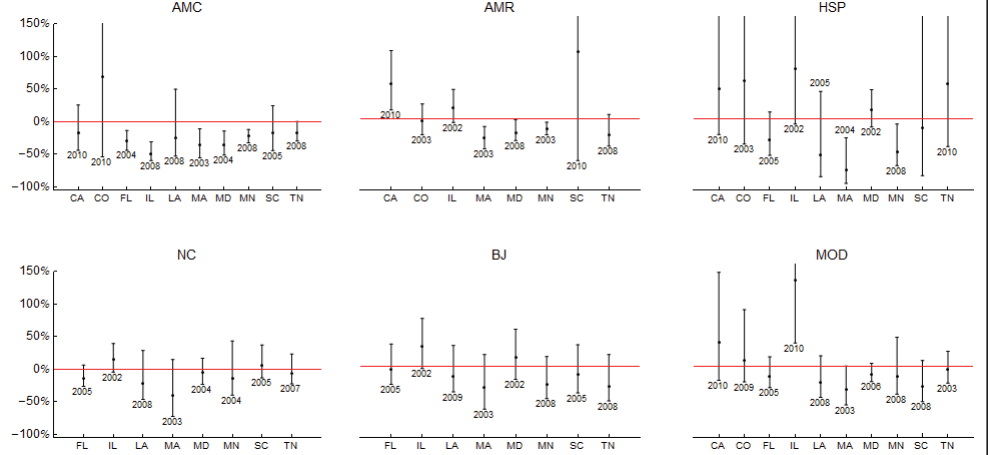
**Largest estimated WNV impact for all bird species and states**. The error bars represent the 95% CIs. The years under the error bars represent the year when the largest impact was observed.

Counts of all species varied considerably over the 17 year study period. American Crows appeared to decline with the emergence of WNV in Illinois (IL), Louisiana (LA), Maryland (MD) and possibly Florida (FL) (Figure 1). In Massachusetts (MA), both American Robins and House Sparrows appeared to decline once WNV emerged (Figure 1). The count trajectories predicted by the model fit the data overall quite well. Most counts were contained in the 95% credible interval associated with the corresponding model prediction (Additional File 1, Figures S1-S6). Among the 54 models examined, more than half (29) explained at least 50% of the total count variance (Table 1). In FL, MA, MD and Minnesota (MN), 25% or more of the variance in American Crow counts was explained by WNV. Similarly high proportions of the variance in the abundance of American Robins was explained by WNV in California (CA), MA, MN and Tennessee (TN). Only in MN, a substantial proportion of the variance in Blue Jay abundance--almost a third--was due to WNV. In IL, WNV accounted for two fifths of the variance in Mourning Doves (Table 1). The impact of WNV on birds varied by species and state. (Figure 2). American Crows suffered a substantial negative impact due to WNV, i.e. 95% credible intervals did not reach above zero, in six of the ten states (FL, IL, MA, MD, MN and TN). The most deleterious effect was found in IL (-58%; 95% CI: -76%, -31%), where in 2010 less than half of the birds that would have been expected in absence of WNV were counted. In MA (2003: -41%; -62%, -13%), MD (2008: -47%; -68%, -18%) and MN (2010: -50%; -67%, -27%) and TN (2010: -33%; -59%, -1%) the largest negative impact was somewhat less dramatic, but still substantial. In most of these cases, with the possible exception of TN, where American Crows appeared to continue their decline in response to WNV, the negative impact of WNV seemed to have stabilized in recent years (not shown). No other species was as consistently affected. American Robins suffered substantially in MA (2010: -50%; -70%, -23%), MD (2008: -23%, -41%, -2%) and MN (2007: -32%, -49%, -13%), but in CA their populations thrived after WNV emergence (2010: 51%; 13%, 100%). House Sparrows were regionally affected, with a more than four fifth decline in MA (2008: -84%; -100%, -32%) and reduction by almost two thirds in MN (2010: -62%, -88%, -8%). Like Northern Cardinals and Blue Jays, Mourning Doves appeared mostly unaffected, but in IL their numbers more than doubled (2010: 126%; 34%, 258%).

**Table 1:** Total variance explained (R-squared) by for all state/bird combinations

	American Crow	American Robin	House Sparrow	Northern Cardinal	Blue Jay	Mourning Dove
CA	56 (4)	77 (39)	15 (7)	-*	-	19 (3)
CO	26 (0)	31 (0)	46 (3)	-	-	19 (0)
FL	58 (30)	-	49 (19)	71 (6)	16 (6)	36 (15)
IL	81 (13)	61 (8)	54 (6)	39 (16)	90 (4)	64 (40)
LA	60 (7)	-	19 (13)	31 (12)	50 (6)	49 (22)
MA	48 (36)	67 (52)	51 (24)	69 (7)	32 (12)	48 (22)
MD	70 (31)	76 (16)	76 (7)	88 (0)	19 (12)	64 (14)
MN	69 (44)	73 (50)	55 (12)	75 (4)	47 (35)	18 (10)
SC	35 (7)	68 (0)	69 (2)	58 (8)	26 (6)	45 (24)
TN	46 (23)	57 (31)	20 (1)	73 (3)	58 (11)	39 (3)

Total variance in percent explained by the model and variance in percent explained by WNNID (in parentheses).

* Species not analyzed for this state.

### Comparison of three methods

For American Crows, we compared our method, hence referred to as autoregressive counterfactual, with two methods that are based on extrapolation from pre-WNV emergence data starting with 1982. The extended time period was necessary to ensure that estimation was based on a similar number of data points used with our method (the extrapolation methods do not use data after WNV emergence for estimation). Because of the extended time period, fewer routes with complete data were available and some states needed to be excluded. Thus, only CA, FL, IL, MA, MD, MN and TN could be used for that comparison. Because of the different time periods used for estimation, r-squared values cannot directly be compared between the autoregressive counterfactual and the extrapolation methods.

#### Proportion of the variance explained

The autoregressive extrapolation method consistently explained a higher proportion of the variance (r-squared) in American Crow counts than the log-linear method. The log-linear and autoregressive extrapolation methods both explained between less than one percent (CA) and almost three fourth or more (MN). The extremely low explanatory power of both extrapolation methods for American Crow counts in CA contrasts with the substantial proportion of the variance explained by the autoregressive counterfactual method. That contrast can be explained by two big count spikes on 1992 and 1994 that are not reflected in the trajectories predicted by both models. Only the second spike was included in the autoregressive counterfactual analysis and was captured quite well by the predicted trajectory. Only in MA (64% and 67%) and MN (74% and 80%) more than half of the variance was explained by the extrapolation models.

#### Impact estimates

**Figure 3:**
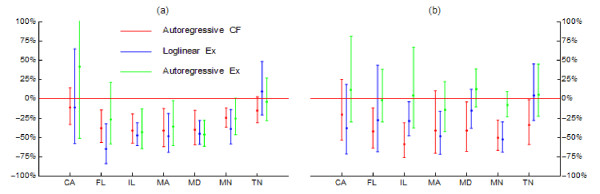
**Comparison of unrestricted impact estimates from different models for American Crows, by state**. Autoregressive CF = Autoregressive counterfactual method; Loglinear Ex = Loglinear extrapolation method; Autoregressive Ex = Autoregressive extrapolation method. Panel (a) represents the year after the highest WNNID incidence in a state; panel (b) represents 2010.

For the year following highest incidence of WNNID, all methods agreed fairly well with respect to large negative impacts (Figure 3a). In IL, for example, the impact on American Crow counts attributed to WNV was -49%, -47% and -43% (autoregressive counterfactual, log-linear extrapolation and autoregressive extrapolation methods) and upper limits of 95% credible intervals calculated using the different methods were below 0. Good agreement was also seen, both in posterior means and credible intervals, in the impact estimates for MD and, to a lesser degree for MA. All methods also agreed that no clear impact could be attributed to WNV in CA and TN. For FL and MN, the autoregressive extrapolation estimate was compatible with no impact (95% credible interval including 0), while the other estimates suggested a substantial negative impact. It is worth noting that relative to both autoregressive estimates, whose posterior means agreed well, the log-linear extrapolation estimates overestimated the negative impact. For 2010, impact estimates obtained by the three methods agreed considerably less, both in posterior means and credible intervals (Figure 3b). According to the estimates derived by the autoregressive extrapolation method, no relevant impact of WNV on American Crows was detected in any state. For MN, on the other hand, impact estimates derived by the autoregressive counterfactual and the log-linear extrapolation method, respectively, were virtually identical. For most other states, with the exception of TN, the two latter methods agreed on a negative impact of WNV on American Crows, but not size or precision of the respective estimates.

## Discussion

We fit a simple first-order autoregressive Poisson model to BBS count data to construct hypothetical count trajectories ("What would counts have been in the absence of WNV?"). These hypothetical (counterfactual) trajectories served to gauge the impact of WNV on North American bird populations. For most bird-state combinations, our model fit annual counts very well (Additional File 1, Figures S1-S6). Up to 90% of the total variance in counts was explained by the model (Blue Jays, IL) and up to 50% of the variance could be attributed to WNV (American Robins, MN). The good fit and the high proportion of variance explained suggests that the model is reasonable.

American Crows appeared to be impacted by WNV particularly severely, which confirms results of previous analyses [26,27] and is consistent with what is known about the sensitivity of that species to WNV [6,8,11-15,17,19-22]. We found a regional negative impact of WNV on American Robins, most pronounced in MA, but also in MD and MN. LaDeau et al. [26] also found that these birds suffered from the emergence of WNV. In most states where a substantial impact of WNV on American Crows was observed, that impact seemed to stabilize (Figure 3). This gives rise to hope that the most vulnerable species may not succumb to WNV. The highly abundant House Sparrow, widely considered a nuisance and destructive to the native avifauna, was noted for the first time to suffer substantial losses due to WNV. This species, likely an important reservoir for WNV, is known to suffer mortality from WNV [28,29]. The lack of detected impact on Blue Jays contrasts with their high susceptibility to WNV infection. However, it confirms a similar observation by LaDeau et al. [26]. No obvious biological or ecological mechanism for this observation can be put forward. A modeling artefact also seems unlikely as inspection of the count trajectories does not reveal any systematic declines coinciding with WNV emergence and transmission. Similarly, Northern Cardinals were not obviously impacted by WNV; this species, too, is vulnerable to lethal WNV infection [30]. These observations are somewhat unsettling, as they imply a lack of understanding of some important aspects of the transmission dynamics of this virus. We included Mourning Doves as a species considered not receptive to WNV infection [20] in response to comments by an anonymous reviewer of an earlier version of this manuscript. As expected, no negative impact of WNV on that species was seen. In the contrary, the species surged after WNV emergence in IL. A biological or ecological explanation for the observed positive association between WNV and Mourning Dove abundance may not be obvious. Yet the possibility of an indirect link should not entirely be discarded [31]. For example, a decreasing abundance of American Crows may lead to lower incidence of nestling and egg predation on Mourning Doves and thus to increased abundance.

We compared impact estimates derived with the autoregressive counterfactual method with estimates obtained by two extrapolation methods. For American Crows, the different methods agreed reasonably well on substantial negative impacts. All three methods estimated that between forty and fifty percent of American Crows in IL had vanished by 2003. Clearly, the agreement between the three methods lends credibility to this assessment. Typically, however, impact estimates that are based on structurally different statistical models will not quantitatively agree. As all these methods rely on hypothetical population trajectories, that are constructed from observational data, we will never be able to empirically verify which method is the most valid. The method should therefore be chosen *a priori*, based on previous knowledge. An additional issue pertinent to any attempt to assess the demographic impact of WNV on birds without directly assessing WNV-associated mortality is the possibility of confounding by factors coinciding with WNV transmission. As pointed out by an anonymous reviewer, variations in climate may, for example, directly and simultaneously affect bird communities and WNV transmission intensity. While analyses such as ours enable us to sensitively detect the potential impact of WNV or other agents on birds on a large geographic and temporal scale, in-depth field studies are needed to confirm and identify these biological and ecological mechanisms. But even if impact estimates derived from such methods are confounded in that manner, as long they are based on a valid model, they will likely represent worst case scenarios, i.e. they quantify the impact under the assumption that it is actually due to WNV and not due to the coincident factor.

An assumption of our analysis is that WNV transmission intensity is proportional to an indicator of WNV transmission such as WNNID. That assumption may be problematic for several reasons. First, human infection is epidemiologically insignificant in the sense that it never results in transmission of WNV (people are dead-end hosts of the virus). Yet, human infection with WNV is a reflection of the enzootic process. But how enzootic transmission affects people depends on many factors, including the seasonal dynamics of different vector mosquitoes. In the American Northeast, for example, *Culex pipiens *mosquitoes, the dominant enzootic and bridge vector there, shift their feeding preference from bird to mammal in late summer [32]. We did not take shifting properties of WNNID as WNV epizootic indicator into account. It is unclear how that phenomenon might be reflected cumulative annual incidence of WNNID. Furthermore, increasing immunity in people could reduce the value of WNNID as indicator for transmission intensity. However, scarce evidence from seroprevalence studies [33,34] indicates that human seroprevalence rarely exceeds 3%, which would likely be insufficient to impact the performance of WNNID as WNV indicator. Second, even though WNV infection, even when not neuroinvasive, is a nationally notifiable disease, reporting may differ by state. As long as the proportion of reported to unreported cases of WNNID does not vary over time, validity of our analysis would not be affected because states are analyzed separately. Third, mosquito-borne transmission is spatially heterogeneous. The extent to which people are affected is also mediated by the proximity of people to transmission foci. A deadly epizootic, leading to massive bird mortality, may not be associated with a spike in WNNID, if it happens away from urban centers with high concentrations of humans and mosquitoes serving as bridge vector. Gingrich et al. [35], for example, documented intense local zoonotic WNV activity in Delaware without a corresponding human epidemic. Even if this is unlikely to happen where *Culex pipiens *and/or *Cx. quinqufasciatus *are the main vector, the spatial scale of our analysis therefore may not adequately reflect the spatial scale of WNV transmission. A local index based on entomological measures (abundance of vector mosquitoes and infection prevalence) would be a much more desirable indicator of transmission intensity. Finally, the demographic impact of WNV on particular avian species may change over time; a detectable prevalence of anti-WNV antibodies in American Crows [36] as well as evidence for an increase of antibody prevalence [37] suggest that possibility. To the extent that this change in susceptibility were substantial, impact estimates might be biased. The good fit of our model with the data, however, supports its validity.

Our methods might be improved by modeling errors that are associated with individual observers. Such errors have the potential to bias population estimates [38] and therefore may be important to include. Other potential model improvements include additional random errors (for example, associated with the change parameter *κ*) and/or modeling of the probability distribution of the WNV indicator variable.

## Conclusions

Despite potential shortcomings our analytic approach adds to previously used methods. Our impact estimates in some key species were qualitatively similar to estimates reported by other authors. American Crows were most consistently negatively affected by WNV, with an estimated population decline of almost 50% in IL. Both American Robins and House Sparrows suffered regional losses, while the other species examined were largely unaffected. Future analysis of the impact of WNV on birds should combine an autoregressive model structure with attempts to model known errors associated with the BBS. Large-scale bird count data is a rich source of information on demographic processes that affect bird populations. However, impact estimates, even if derived from structurally valid statistical models, are prone to confounding if accurate and relevant indicators of enzootic WNV transmission are lacking. Ideally, such estimates should be validated with direct field observation.

## Methods

### Data

We obtained BBS raw bird count data online from the North American Breeding Bird Survey http://www.pwrc.usgs.gov/bbs/index.html for each of ten states, representing all major regions of the continental United States (in alphabetical order): California (CA), Colorado (CO), Florida (FL), Illinois (IL), Louisiana (LA), Massachusetts (MA), Maryland (MD), Minnesota (MN), South Carolina (SC) and Tennessee (TN). The survey is a collaboration between the U.S. Geological Survey's Patuxent Wildlife Research Center and the Canadian Wildlife Service's National Wildlife Research Centre. Data were obtained on five common North American birds that are known to suffer high mortality from WNV and/or are known to be important amplification hosts of the virus: American Crows (*Corvus brachyrhynchos*), American Robins (*Turdus migratorious*), Blue Jays (*Cyanocitta cristata*), House Sparrows (*Passer domesticus*) and Northern Cardinals (*Cardinalis cardinalis*). Mourning Doves (*Zenaida macroura*) were included as a species that is thought to be unaffected by WNV. Annual counts of American Robins were not analyzed for LA and FL because they were consistently below ten. For both Blue Jays and Northern Cardinals, counts from California and Colorado were not analyzed because they were either consistently below ten or entirely missing. For each species/year combination considered, the sum of all counts from routes with complete data for the years 1994 through 2010 (no missing years) was analyzed. On average, 14 routes were analyzed per state, ranging from five (CO) to 32 (IL), totaling 140 routes.

We used state annual rates of WNV neuroinvasive disease (WNNID) cases as indicators for cumulative transmission intensity of WNV. WNNID reports were obtained from the yearly human case count archives from the CDC WNV Statistics, Surveillance and Control website http://www.cdc.gov/ncidod/dvbid/westnile/index.htm. The data were obtained from the Encephalitis/Meningitis category in each yearly CDC summary. To obtain rates we divided these numbers by the state mid-year population estimate for that year (U.S. Census Bureau http://www.census.gov/popest/states/NST-ann-est.html).

El Niño-Southern Oscillation (ENSO) has been found to explain up to 90% of the annual variation in the productivity of land-breeding birds [39]. We therefore included a dichotomous ENSO variable into our models. The data were obtained from the National Oceanographic and Atmospheric Association (NOAA) and each year in which "abnormal" warming (based on a +0.5°C threshold of the Oceanic Niño Index relative to the 1971-2000 base period [40]) began and continued through the next year was considered an El Niño year http://www.cpc.ncep.noaa.gov/products/analysis_monitoring/ensostuff/ensoyears.shtml.

### Data analysis

The following model was fit to annual counts of the six bird species. Each model was separately fit to time series of counts for all existing bird/state combinations. The annual counts *x_t_*, for *t *∈ 2, ..., 17, for each existing bird/state combination are assumed to be distributed according to Poisson variables whose parameters *λ_t _*vary around *μ_t_*, i.e.(1)

Through the parameters *μ_t_*, these counts are modeled as simple first-order autoregressive process [41], i.e.(2)

This type of model will be referred to as an autoregressive counterfactual model. The model is similar to the one proposed by [42] for interspecific demographic interactions. The parameter *κ_t_*, for *t *∈ 2, ..., 16, represents the annual net change of the population from year *t - *1 to *t*. For each bird/state combination, *κ_t _*was estimated based on the following model:(3)

where *β*_0 _represents the baseline change, *y_t _*is the WNNID incidence rate for year *t *and *z_t _*represents the ENSO variable (1 = abnormal warming, 0 = no abnormal warming). Both variables were allowed to have a lagged effect (*y*_*t - *1 _and *z*_*t - *1_, respectively).

Models were fit using a Markov chain Monte Carlo (MCMC) algorithm implemented in WinBUGS (Imperial College and Medical Research Council, UK) [43]. Most parameters were *a priori *assumed to be distributed according to N(0, 10^3^), which corresponds to essentially flat priors over their plausible range (Additional file 2). The empirical posterior distributions of the parameters were obtained from 30,000 MCMC samples, resulting from three chains with 200,000 burn-in iterations and 10,000 samples each (Additional File 2). As determined by visual inspection of chain histories, all models converged. Posterior means and 95% credible intervals (CIs) were calculated for all parameters of interest.

We estimated the proportion of the total variance explained by final models using a variant of the coefficient of determination or r-squared [44] that was based on the posterior mean of *μ*, rather than *λ*, because overdispersion forces the model to closely reproduce the data, giving rise to r-squared values close to one. This calculation was performed for each MCMC iteration. To quantify the proportion of the variance explained by WNNID, we fit also models without WNNID terms. To calculate impact estimates we constructed hypothetical population trajectories , for *t *∈ *M*, ..., *N *, starting with a state's first year of WNNID reports, *M*. These trajectories are defined by hypothetical expected counts that would be observed in the absence of WNV transmission:

and

Accordingly, κ* is the counterfactual change parameter (no WNNID terms).

These impact estimates will be referred to as autoregressive counterfactual.

We compared the performance of our method with two extrapolation methods. The first of these methods is based on a log-linear model of the counts and is similar to the model used by LaDeau et al. [26]. The log-linear model of the counts has the following form:(4)

where *α*_1 _is a log-linear trend, centered at t*, the mid-point of the data period to which the model was fit and *ε_t _*is a N(0, *τ*) error term. Impact estimates were based on hypothetical count trajectories that were calculated from the linear combinations of covariate values and parameter estimates for the years after WNV emergence. From the resulting hypothetical counts we subtracted the Poisson parameter estimates of the actual counts and divided the result by the hypothetical counts to obtain a proportional impact measure (log-linear extrapolation).

Similarly, autoregressive models (2 and 3, but without WNNID terms) were fit only to pre-emergence counts. Calculation of impact estimates (autoregressive extrapolation) were performed as described for the log-linear model.

The comparison was done using American Crow data. As the extrapolation methods do not use data after WNV emergence for parameter estimation, we included data from 1982 until WNV emergence, resulting in 29 (rather than 17) years of data. As a consequence of using only routes with complete data, fever routes were available for this analysis and three states could not be analyzed. For seven states (CA, FL, IL, MA, MD, MN and TN) data were available for the comparison of impact estimates. Impact estimates were compared for the year after the highest WNV activity (as measured by WNNID incidence) and for 2010.

## Authors' contributions

IMF designed the study, developed methods, analyzed the data and wrote the manuscript. RHB prepared the data, analyzed the data and contributed to the manuscript. IHM provided ornithological expertise and contributed to the manuscript. All authors read and approved the final manuscript.

## Supplementary Material

Additional file 1 **This zipped folder contains the supplemental Figures (Figure 1 suppl.pdf through Figure 6 suppl.pdf) and legends (Supplemental Figure legends 2.pdf)**. Click here for file

Additional file 2 **This zipped folder contains a sample data file (datamatnew_amcIL.dat), WinBUGS code (model 2 a1a2d1d2.txt), R code (calculations AMC IL.txt) and a description of these files (Description of suppl files 2.doc)**. Click here for file
