# Supplementary material for: The impact of West Nile virus on the abundance of selected North American birds
Source: BMC Vet Res. 2011 Aug 11;7:43. doi: 10.1186/1746-6148-7-43 (PMC3163188; doi:10.1186/1746-6148-7-43)
Supplement: Additional file 1 — This zipped folder contains the supplemental Figures (Figure 1 suppl.pdf through Figure 6 suppl.pdf) and legends (Supplemental Figure legends 2.pdf). [file 1746-6148-7-43-S1.ZIP › Supplemental Figure legends 2.pdf]

Supplemental Figure Legends

Figure 1. Observed and fitted counts of American Crows (*Corvus brachyrhynchos*) and annual WNNID reports, by state and WNNID incidence. Red lines: Predicted counts, connected with line; Pink area: Area between the lines connecting the upper and lower 95% credible interval limits, respectively, of the number of counts predicted by the model; Light blue area: Area between the lines connecting the upper and lower 95% credible interval limits of the counts predicted by the model in absence of WNV; Red bars: WNNID incidence. The primary y–axis refers to the observed counts (grey circles) and the secondary y–axis represent annual incidence of WNNID (per 1,000,000 population).

Figure 2. Observed and fitted counts of American Robins (*Turdus migratorius*) and annual WNNID reports, by state.

Figure 3. Observed and fitted counts of House Sparrows (*Passer domesticus*)and annual WNNID reports, by state.

Figure 4. Observed and fitted counts of Northern Cardinals (*Cardinalis cardinalis*)and annual WNNID reports, by state.

Figure 5. Observed and fitted counts of Blue Jays (*Cyanocitta cristata*) and annual WNNID reports, by state.

Figure 6. Observed and fitted counts of Mourning Doves (*Zenaida macroura*) and annual WNNID reports, by state.
