# Supplementary figures and images for: The impact of West Nile virus on the abundance of selected North American birds
Source: BMC Vet Res. 2011 Aug 11;7:43. doi: 10.1186/1746-6148-7-43 (PMC3163188; doi:10.1186/1746-6148-7-43)

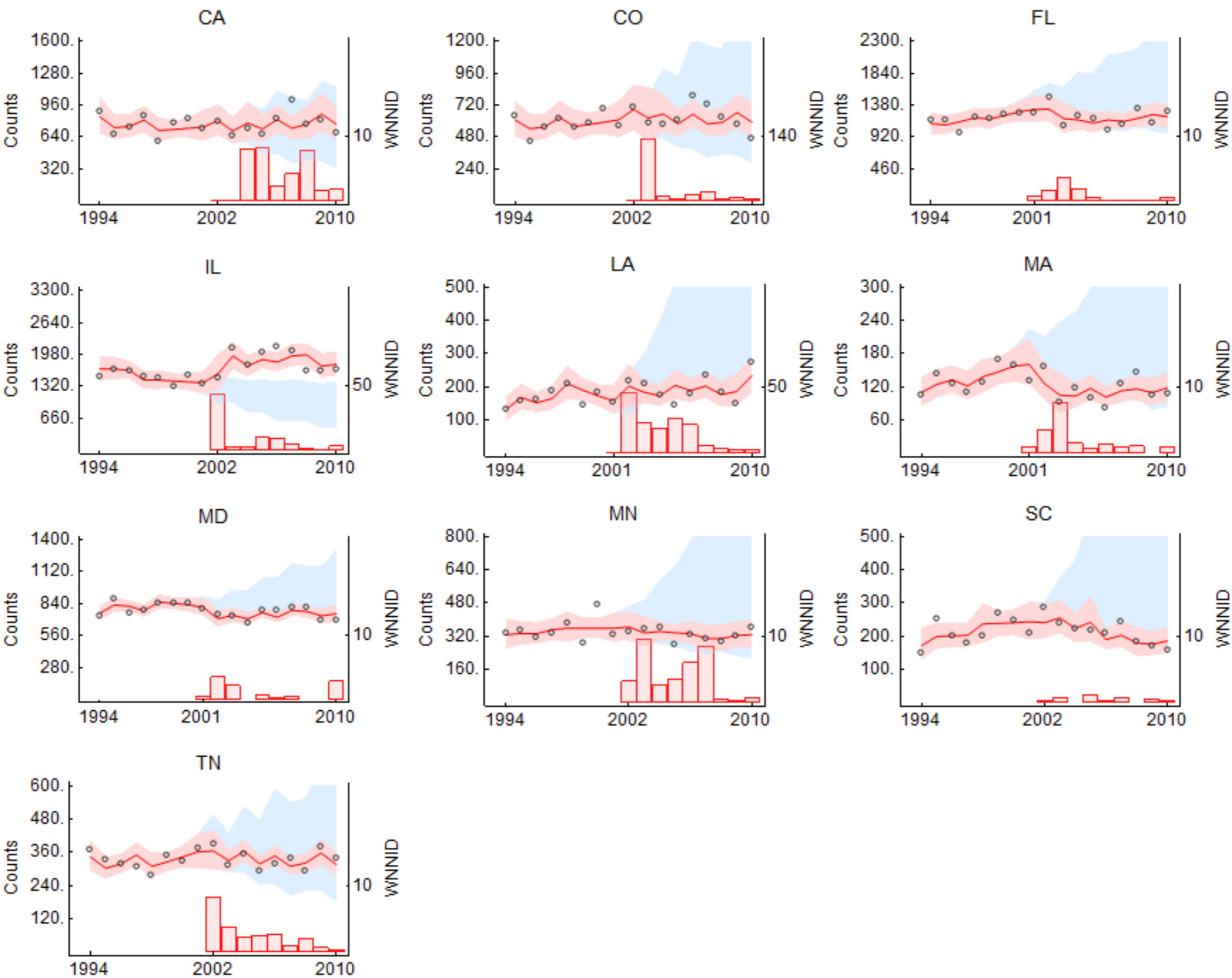

Supplement: Additional file 1 — This zipped folder contains the supplemental Figures (Figure 1 suppl.pdf through Figure 6 suppl.pdf) and legends (Supplemental Figure legends 2.pdf). [file 1746-6148-7-43-S1.ZIP › Figure 6 suppl.pdf]

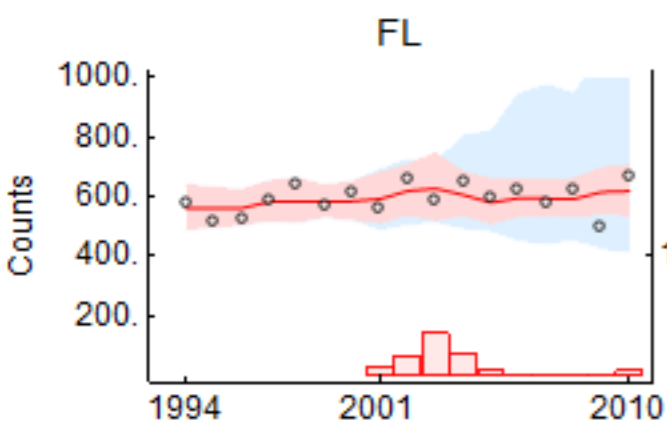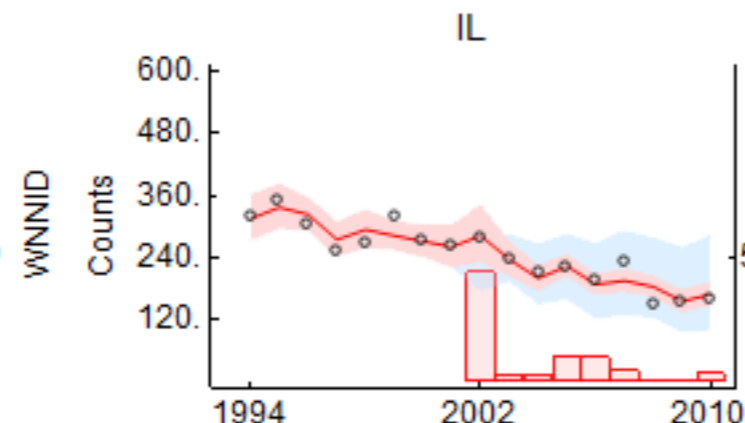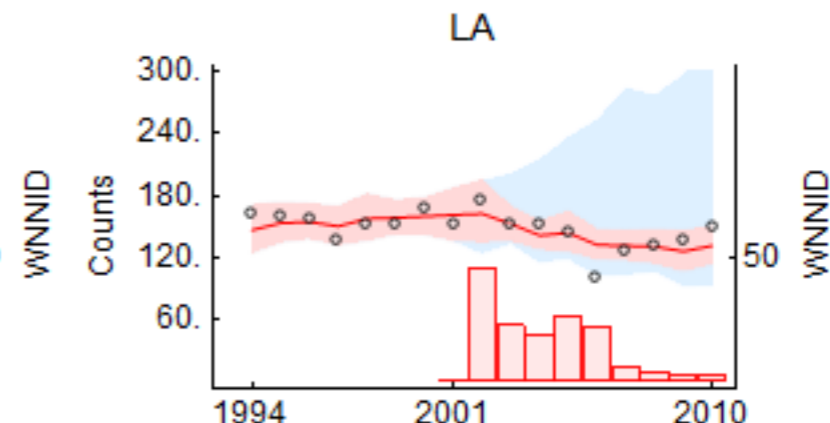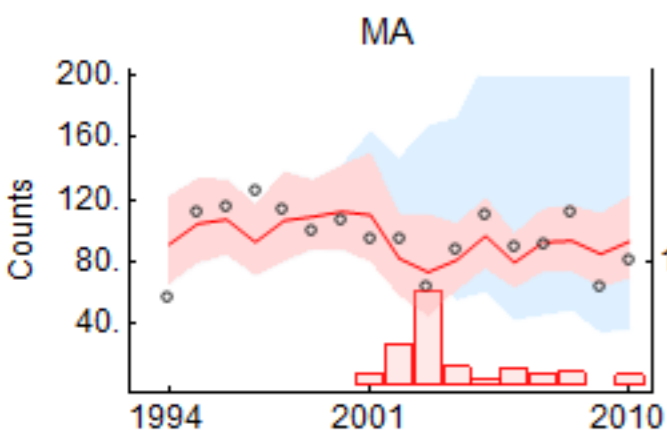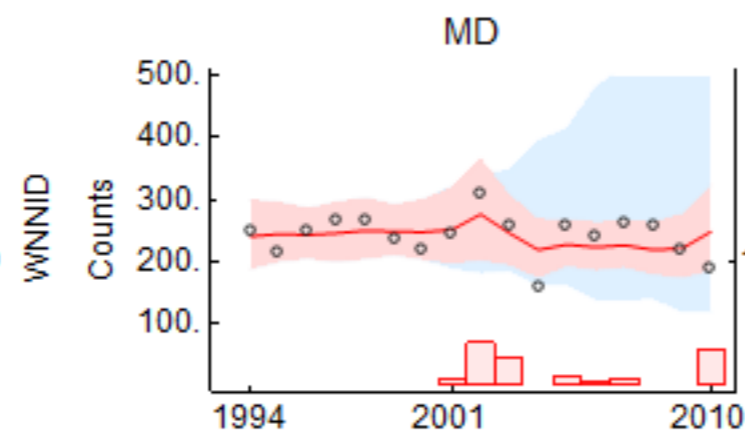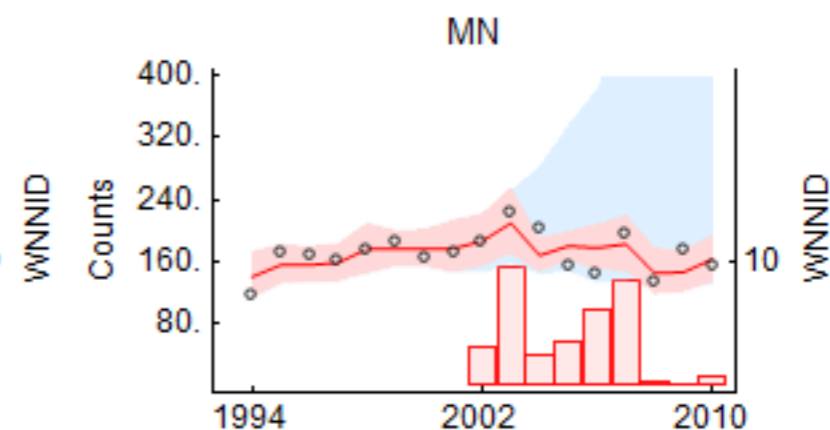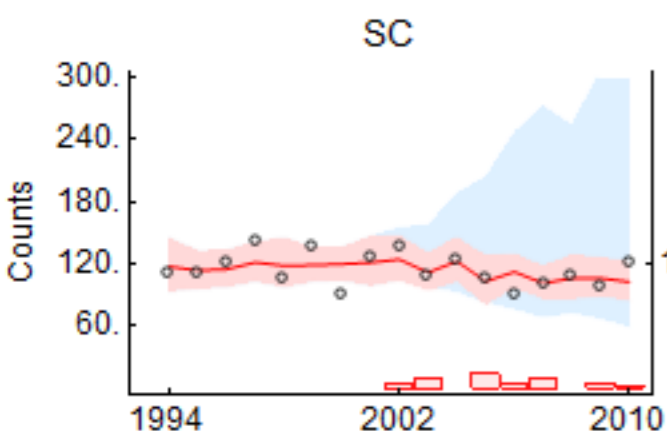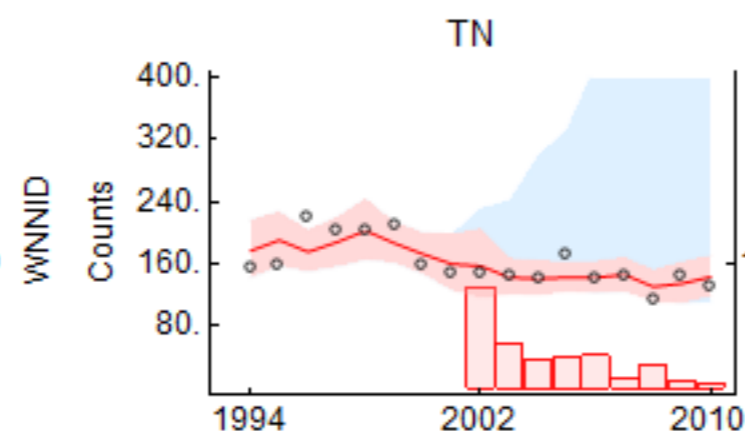

Supplement: Additional file 1 — This zipped folder contains the supplemental Figures (Figure 1 suppl.pdf through Figure 6 suppl.pdf) and legends (Supplemental Figure legends 2.pdf). [file 1746-6148-7-43-S1.ZIP › Figure 5 suppl.pdf]

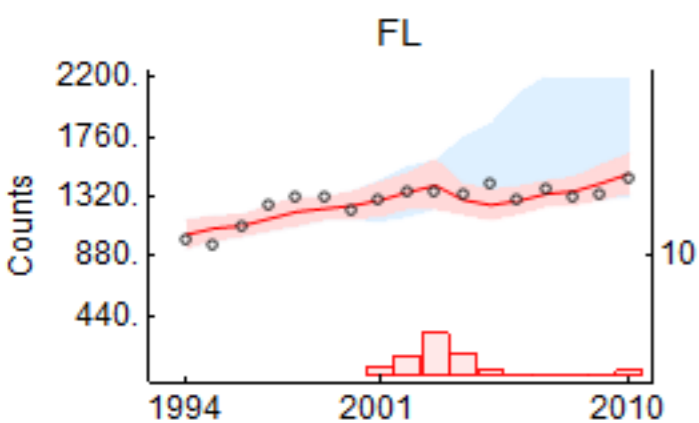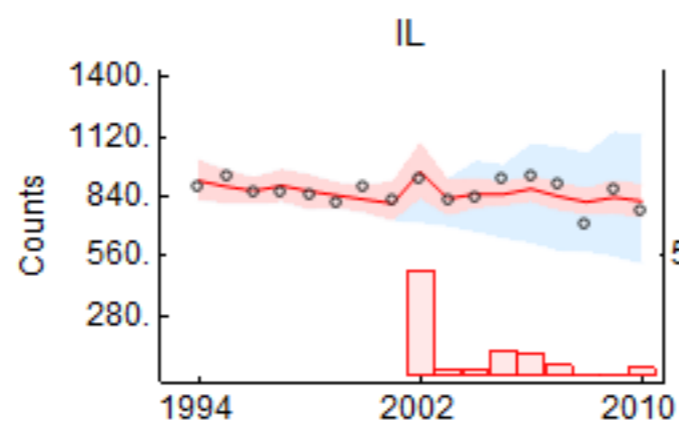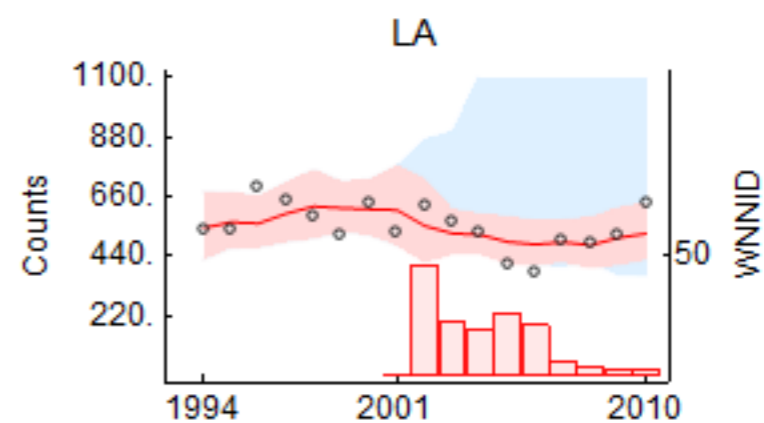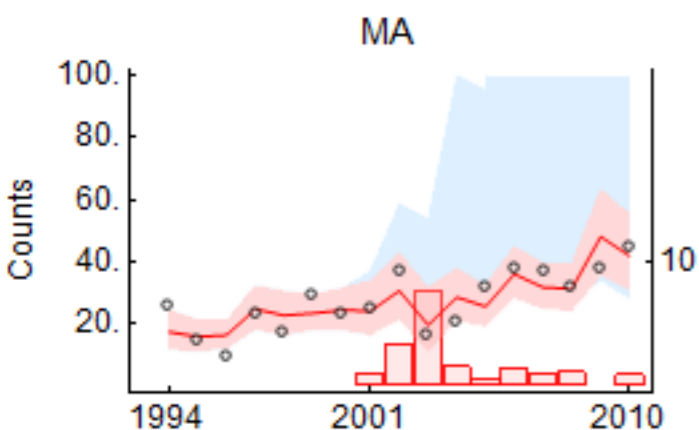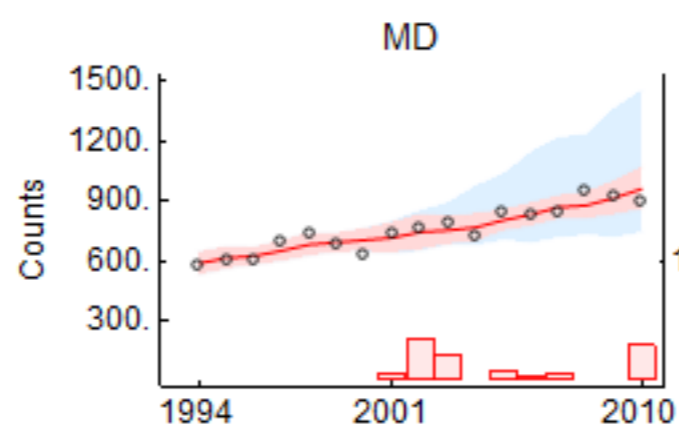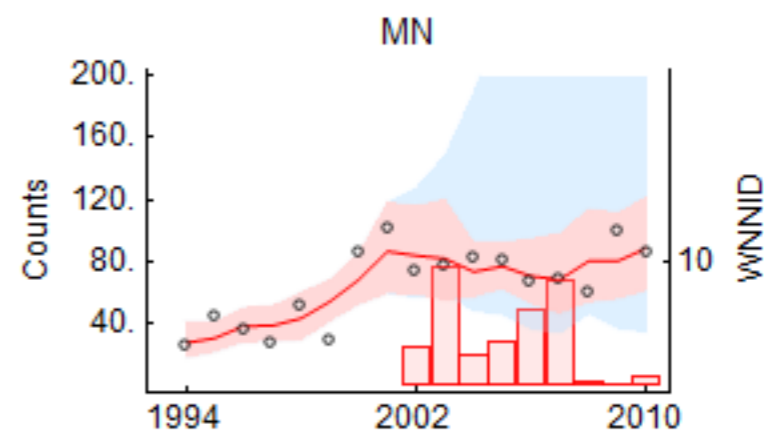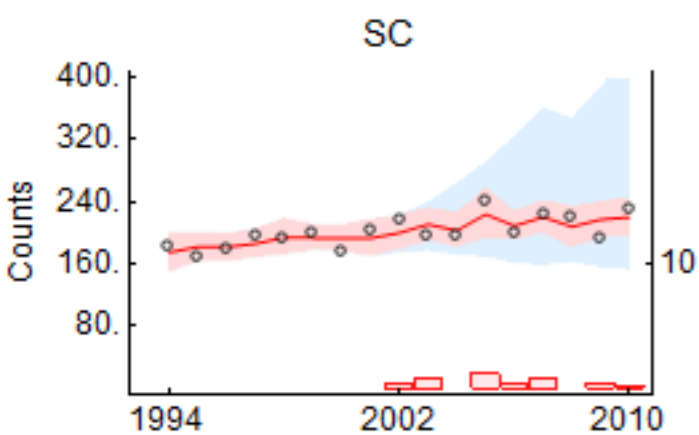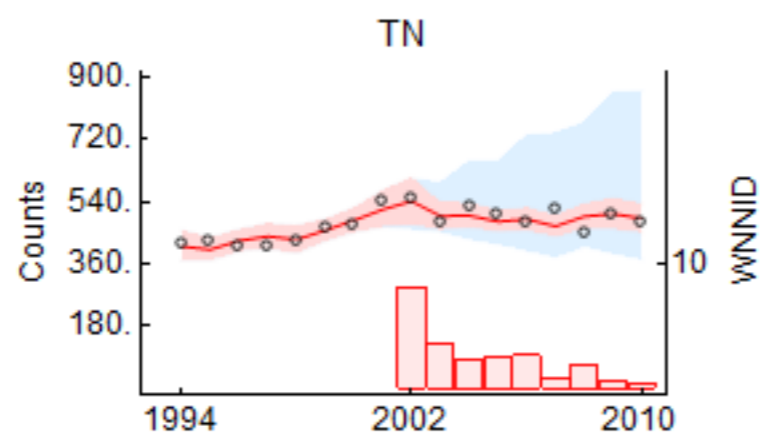

Supplement: Additional file 1 — This zipped folder contains the supplemental Figures (Figure 1 suppl.pdf through Figure 6 suppl.pdf) and legends (Supplemental Figure legends 2.pdf). [file 1746-6148-7-43-S1.ZIP › Figure 4 suppl.pdf]

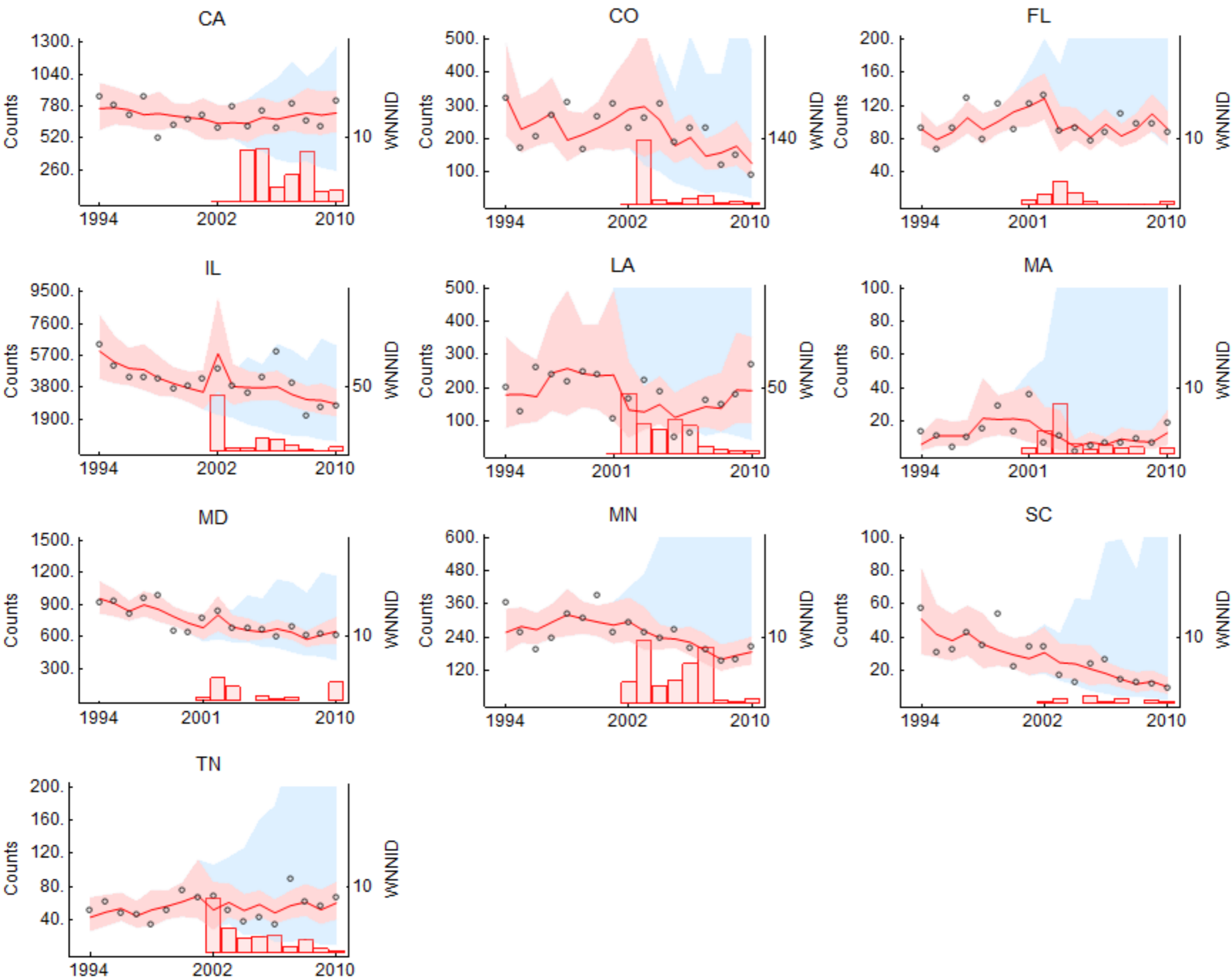

Supplement: Additional file 1 — This zipped folder contains the supplemental Figures (Figure 1 suppl.pdf through Figure 6 suppl.pdf) and legends (Supplemental Figure legends 2.pdf). [file 1746-6148-7-43-S1.ZIP › Figure 3 suppl.pdf]

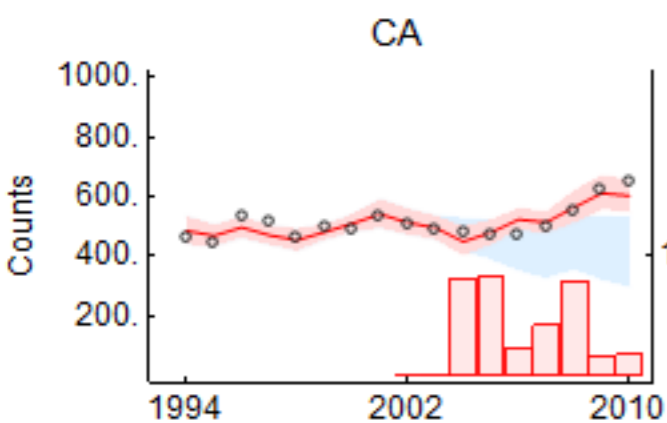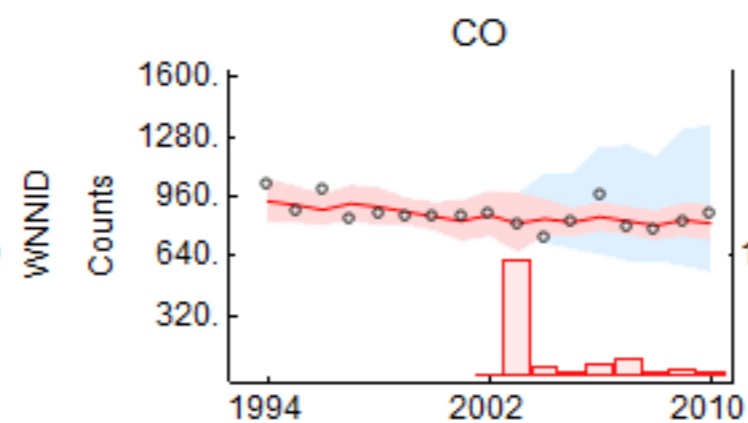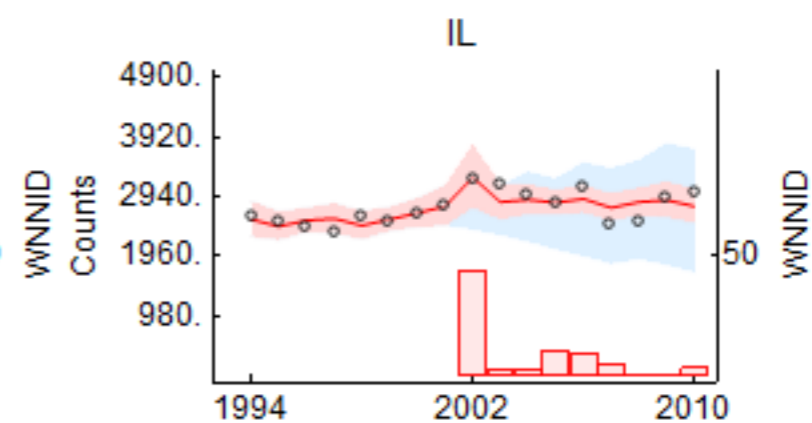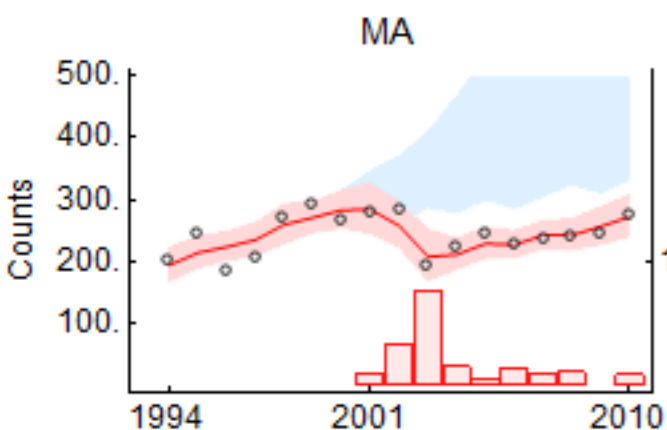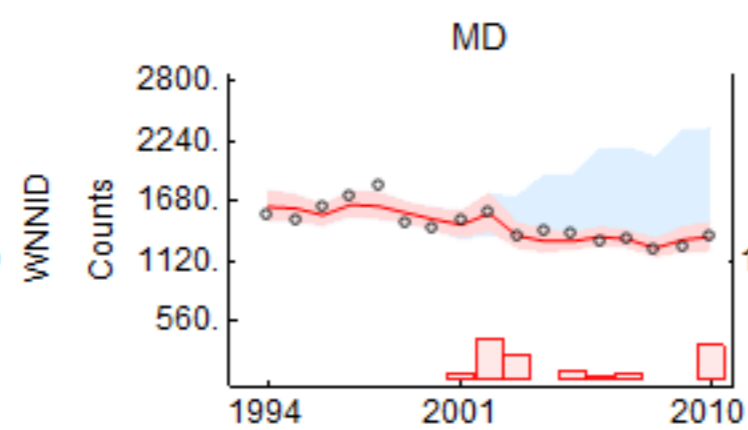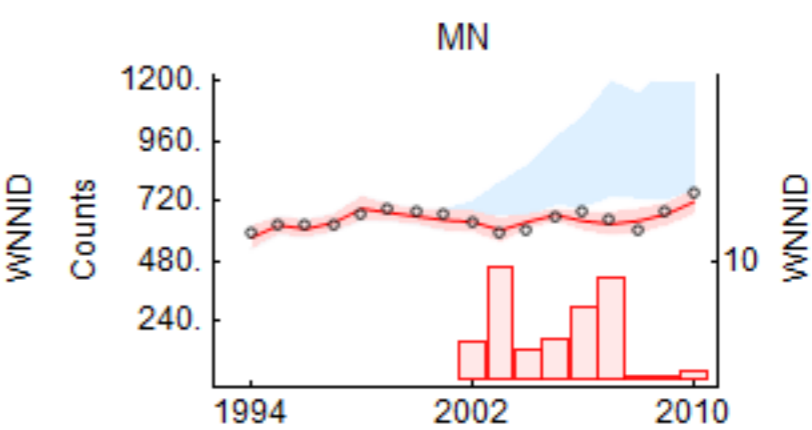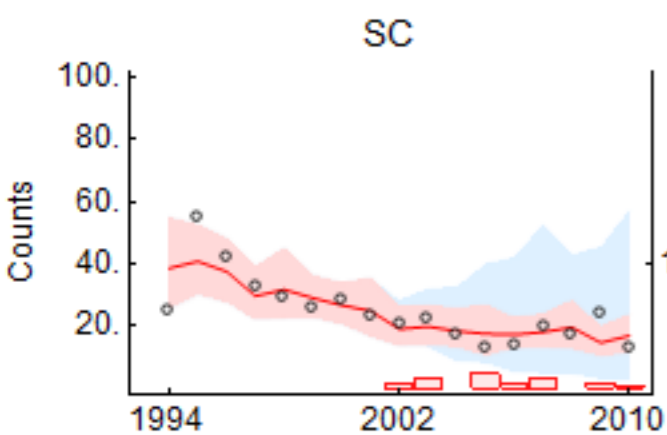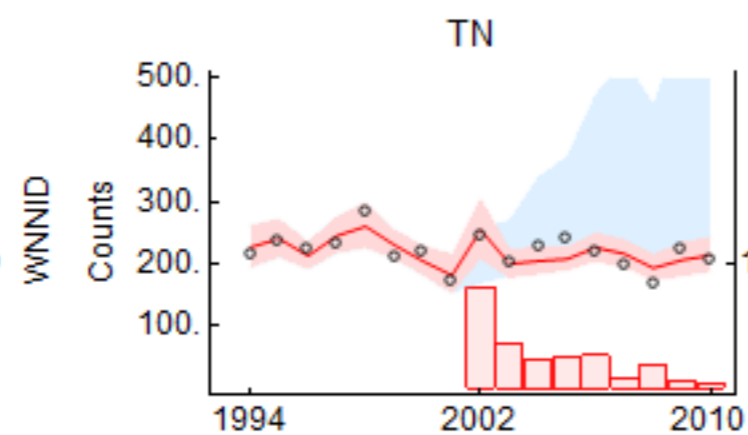

Supplement: Additional file 1 — This zipped folder contains the supplemental Figures (Figure 1 suppl.pdf through Figure 6 suppl.pdf) and legends (Supplemental Figure legends 2.pdf). [file 1746-6148-7-43-S1.ZIP › Figure 2 suppl.pdf]

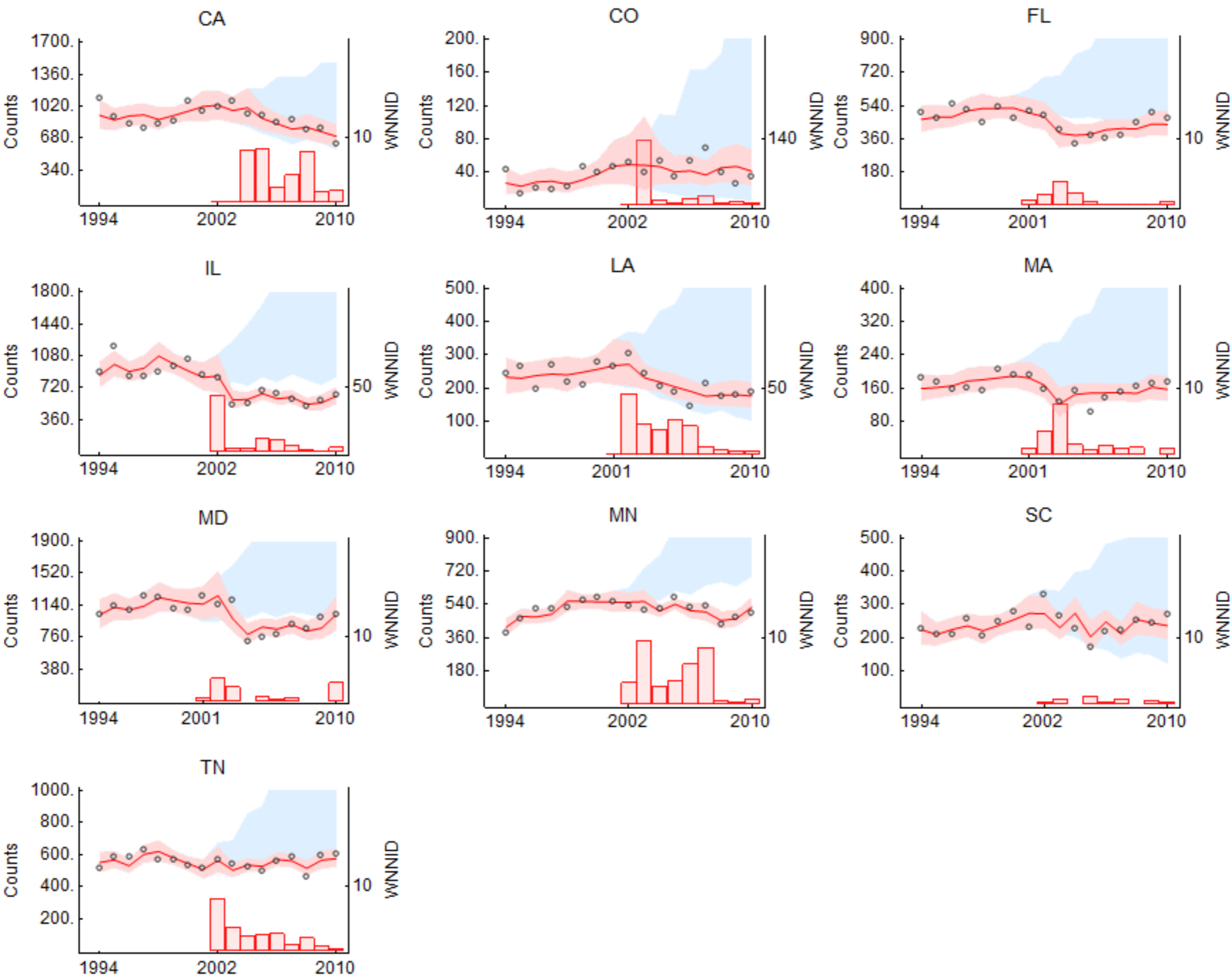

Supplement: Additional file 1 — This zipped folder contains the supplemental Figures (Figure 1 suppl.pdf through Figure 6 suppl.pdf) and legends (Supplemental Figure legends 2.pdf). [file 1746-6148-7-43-S1.ZIP › Figure 1 suppl.pdf]
