# Supplementary material for: The impact of West Nile virus on the abundance of selected North American birds
Source: BMC Vet Res. 2011 Aug 11;7:43. doi: 10.1186/1746-6148-7-43 (PMC3163188; doi:10.1186/1746-6148-7-43)
Supplement: Additional file 2 — This zipped folder contains a sample data file (datamatnew_amcIL.dat), WinBUGS code (model 2 a1a2d1d2.txt), R code (calculations AMC IL.txt) and a description of these files (Description of suppl files 2.doc). [file 1746-6148-7-43-S2.ZIP › Description of suppl files 2.doc]

This document describes the documents that are uploaded in a zipped folder

datamatnew_amcIL.dat

Dataset contains the counts (cnts) of American Crows for the years (yr) 1994 to 2009 from 37 routes with complete data; wnnid: number of annual WNNID disease reports; en: ENSO event; pop: mid-year population estimate.

model 2 a2d1d2.txt

WinBUGS model

calculations AMC IL.txt

R code to run the analysis for American Crows in IL
